# The caudal bursa in the Heligmonellidae (Nematoda: Trichostrongylina). Characterization and hypothesis on its evolution

**DOI:** 10.1051/parasite/2012191003

**Published:** 2012-02-15

**Authors:** M.C. Durette-Desset, M.C. Digiani

**Affiliations:** 1 Département de Systématique et Evolution, Muséum national d’Histoire naturelle, UMR 7138 associée au CNRS CP 52 61, rue Buffon 75231 Paris Cedex 05 France; 2 CONICET. División Zoología Invertebrados, Museo de La Plata, Paseo del Bosque s/n 1900 La Plata Argentina

**Keywords:** Nematoda, Heligmonellidae, bursal pattern, bursal symmetry, evolution, Nematoda, Heligmonellidae, pattern boursal, symétrie boursale, évolution

## Abstract

The different patterns of the caudal bursa of the Heligmonellidae (Nematoda) are redefined, taking into account the grouping of rays 2-6 and the sequence of origin of these rays from their common trunk. The type of symmetry of the caudal bursa is also redefined. The following patterns were observed and characterized: the basic patterns: types 2-3, 2-2-1, 1-3-1 and 1-4 and the intermediary patterns: type 2-3 tending to type 2-2-1, type 2-2-1 tending to type 1-3-1, type 1-3-1 tending to type 1-4 and type 2-2-1 tending to type 1-4. An evolutionary interpretation of the patterns is attempted and seems to follow the direction: 2-3 to 2-2-1 to 1-3-1 to 1-4. Seven atypical patterns are described. The caudal bursae were classified based on their symmetry: subsymmetrical, dissymmetrical and asymmetrical. Independently of the type of symmetry, the two latero-ventral lobes may have the same or different patterns. The type of symmetry, the ratio between the two latero-ventral lobes and a characteristic pattern were utilized to characterize the caudal bursae at the level of the genus and the subfamily. The combination of the right/left ratio and the type of symmetry gives heterogeneous results, with no real association between these characters. The most conspicuous asymmetries and dissymmetries were found among the Nippostrongylinae. The most frequent pattern in the Heligmonellidae is the basic type 2-2-1; types 1-3-1 and 1-4 are less frequent but are characteristic of several genera; type 1-4 is absent from the Heligmonellinae. Whatever the pattern, in the Heligmonellidae rays 4 and 5 are the last to diverge from the common trunk of rays 2-6.

## Abstract (fr)

Les différents patterns de la bourse caudale chez les Heligmonellidae (Nematoda) sont redéfinis en tenant compte du groupement des côtes 2-6 et de la séquence d’apparition de ces côtes sur leur tronc commun. Le type de symétrie est également redéfini. Les patterns suivants sont observés et caractérisés : les patterns de base : type 2-3, 2-2-1, 1-3-1 et 1-4 et les patterns intermédiaires : type 2-3 à tendance 2-2-1, 2-2-1 à tendance 1-3-1, 1-3-1 à tendance 1-4 et 2-2-1 à tendance 1-4. Une interprétation évolutive des patterns est proposée et semble suivre la direction : 2-3 vers 2-2-1 vers 1-3-1 vers 1-4. Sept patterns atypiques sont décrits. Les bourses caudales sont classifiées selon leur symétrie en : subsymétrique, dissymétrique et asymétrique. Indépendamment du type de symétrie, les deux lobes latéro-ventraux peuvent avoir un pattern identique ou différent. Le type de symétrie, le rapport évolutif entre les deux lobes latéro-ventraux et un type de pattern caractéristique ont été utilisés pour caractériser les bourses caudales au niveau du genre et de la sous-famille. La combinaison du rapport droite/gauche et le type de symétrie donnent des résultats hétérogènes sans véritable lien entre ces caractères. Les asymétries et les dissymétries les plus manifestes sont trouvées parmi les Nippostrongylinae. Le pattern le plus fréquemment rencontré chez les Heligmonellidae est le type de base 2-2-1 ; les types 1-3-1 et 1-4 sont moins fréquents mais caractéristiques de plusieurs genres ; le type 1-4 est absent chez les Heligmonellinae. Chez les Heligmonellidae, quel que soit le pattern, les côtes 4 et 5 sont les dernières à diverger du tronc commun aux côtes 2-6.

## Introduction

**Fig. 1.**
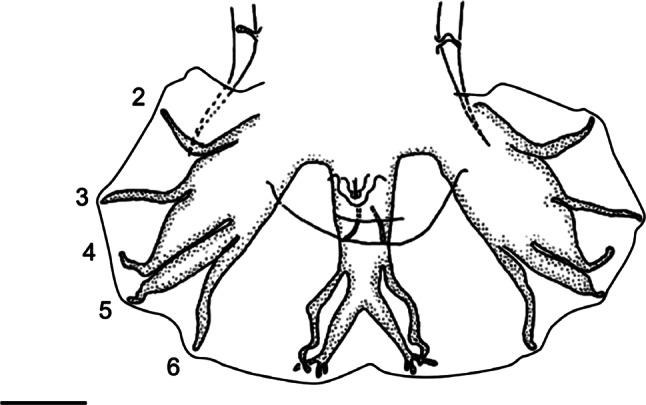
Numbering of rays 2 to 6 according to Chabaud *et al.* (1970). Rays 2 and 3: ventral rays. Ray 2: ventro-ventral ray. Ray 3: lateroventral ray. Rays 4-6: lateral trident. Ray 4: externo-lateral ray. Ray 5: medio-lateral ray. Ray 6: postero-lateral ray. Ex: *Pudica gonosoma* Cassone & Durette-Desset, 1991. After Cassone & Durette-Desset (1999), modified. Scale-bar: 50 μm.

The Trichostrongylina have a common origin with the other suborders of the order Strongylida, arising from an ancestor close to the Rhabditida (Durette-Desset *et al.*, 1994; Blaxter *et al.*, 1998, 2001). In the strongylid nematodes, unlike the Rhabditida, the tail of the male widens in order to form a caudal bursa made up of two latero-ventral lobes and one dorsal lobe. Therefore the caudal bursa is a derived character regarded as a synpomorphy for the Strongylida. Durette-Desset & Chabaud (1981) and Durette-Desset (1985), proposed a classification of the Trichostrongyloidea which they divided into three supra familiar groups: the “Trichostrongylids”, the “Molineids” and the “Heligmosomids”. In these classifications, they highlighted various types of caudal bursae, based mainly on the grouping of rays 2 to 6 (*i.e.* the rays supporting the latero-ventral lobes) (Fig. 1). In 1981, four patterns were defined: 1-3-1, characteristic of the “Trichostrongylids”, 2-1-2, characteristic of the “Molineids” and 2-2-1 and 3-2, characteristic of the “Heligmosomids”. Durette-Desset (1985) added type 2-3 and specified that each grouping is characteristic of a given evolutionary line or a given family. Then, Durette-Desset & Chabaud (1993) raised the Trichostrongyloidea to a suborder: the Trichostrongylina and each supra familiar group became a superfamily: the Trichostrongyloidea, the Molineoidea and the Heligmosomoidea.

This descriptive system however seems to us to be insufficient to properly describe the caudal bursae of some Nippostrongylinae, especially the genera such as *Heligmonina* Baylis, 1928 and *Stilestrongylus* Freitas, Lent & Almeida, 1937, which do not show the same pattern in each lateral lobe. Within the framework of a revision of the Heligmonellidae, the aim of this work is to redefine the different patterns of the caudal bursa found within this family, taking into account not only the grouping of rays 2-6 but also the sequence of origin of these rays from their common trunk. The type of symmetry of the caudal bursa is also redefined. This enables us not only to make the descriptions of the caudal bursae more accurate, but also to highlight the characteristic type(s) of pattern within each genus and to attempt an evolutionary interpretation of the patterns.

## Materials and Methods

The elements usually considered in the description of a caudal bursa (CB) are: symmetry, pattern of latero-ventral lobes, development of the dorsal lobe and characters of the dorsal ray and rays 8. The characters of the dorsal ray and rays 8 are not treated in this work. Some terms concerning the symmetry and the pattern of the latero-ventral lobes are redefined as follows.

### Types of symmetry

**Fig. 2:**
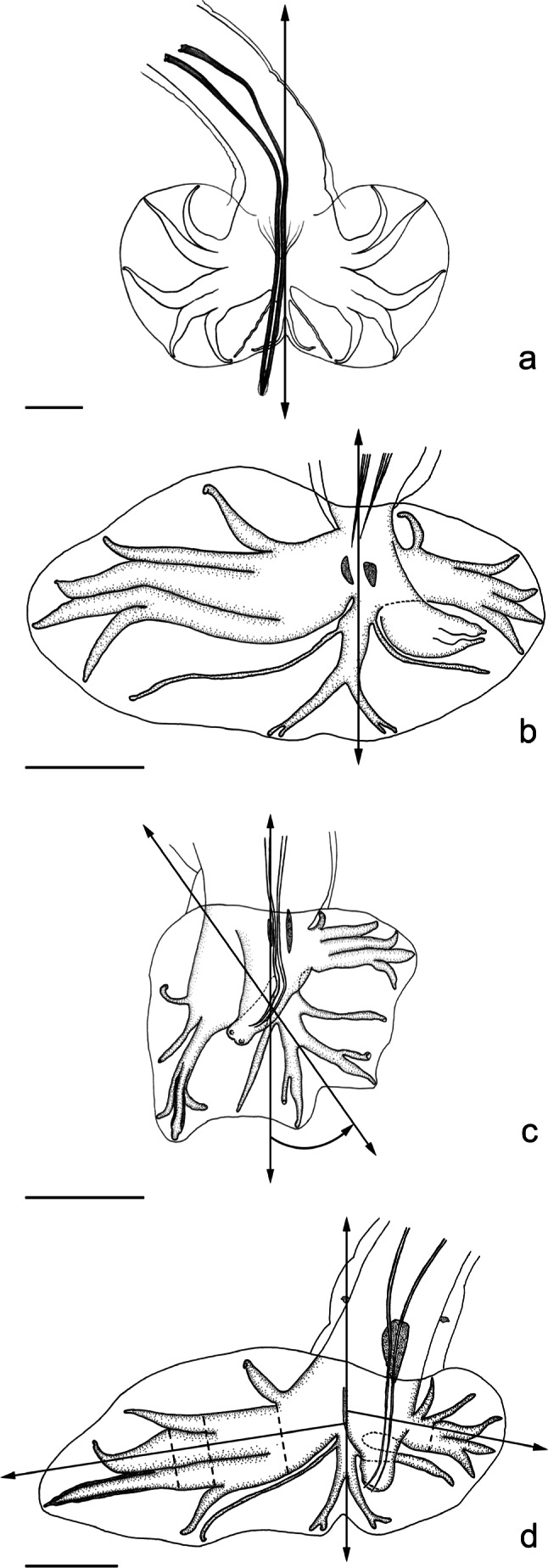
**Figs. 2a-c** Types of symmetry of the caudal bursa. a. Subsymmetrical: both lobes are of similar size and shape in relation to the axis of the dorsal ray, which passes through the sagittal axis of the worm. Ex: *Pudica gamma* (Travassos, 1918). After Travassos (1921), modified. b. Dissymmetrical: one lobe is better developed in relation to the axis of the dorsal ray, which passes through the sagittal axis of the worm. Ex: *Malvinema scapteromy*s (Suriano & Navone, 1996). In this case, it involves the right lobe. After Digiani *et al.* (2003), modified. c. Asymmetrical: the axis of the dorsal ray does not pass through the sagittal axis but is displaced to the right or left side of the worm. Both lobes may or may not have the same degree of development with respect to the axis of the dorsal ray. Ex: *Malvinema victoriae* Digiani, Sutton & Durette-Desset (2003). In this case, the right lobe is slightly better developed. After Digiani *et al.* (2003), modified. **Fig. 2d** Determination of the point of divergence of rays 2 to 6 from their common trunk. The levels of divergence of rays 2 to 6 from the common trunk are indicated by the dotted lines, which are perpendicular to a main axis represented by a straight line passing through ray 4. Ex: *Malvinema carolinae* Digiani, Sutton & Durette-Desset (2003). In the right lobe, ray 2 is the first (the most proximal ray) to diverge from the common trunk; the point of divergence of ray 3 is distal to that of ray 6. Rays 4 and 5 are the last to diverge. After Digiani *et al.* (2003), modified. Scale-bars Figs. 2a–d: 50 μm.
- Dissymmetrical bursae (CB DS): One lobe is larger than the other in relation to the axis of the dorsal ray, which passes through the sagittal axis of the worm. When the right lobe (RL) is larger it is cited as CB DS RL+. When the left lobe (LL) is larger it is cited as CB DS LL+ (Fig. 2b).
- Asymmetrical bursae (CB AS): The axis of the dorsal ray does not pass through the sagittal axis but is displaced to the right or the left side of the worm. One lobe may or may not be larger than the other in relation to this axis. When the right lobe (RL) is larger it is cited as CB AS RL+. When the left lobe (LL) is larger it is cited as CB AS LL+ (Fig. 2c).

There are three types, which concern the degree of development of the latero-ventral lobes in relation to the sagittal axis of the worm:

- Subsymmetrical bursae (CB SS): both lobes are of similar size and shape in relation to the axis of the dorsal ray, which passes through the sagittal axis of the worm (Fig. 2a).

### Types of pattern

The main features considered in the definition of the patterns are the grouping of the rays and the sequence of origin (or divergence) of these rays from their common trunk. The level of divergence of a ray from a common trunk (rays 2 to 6, 3 to 5 or 3 to 6) is calculated on a straight line represented by ray 4; at the level of divergence another line is traced perpendicularly to the first one, as shown in Fig. 2d.

**Fig. 3:**
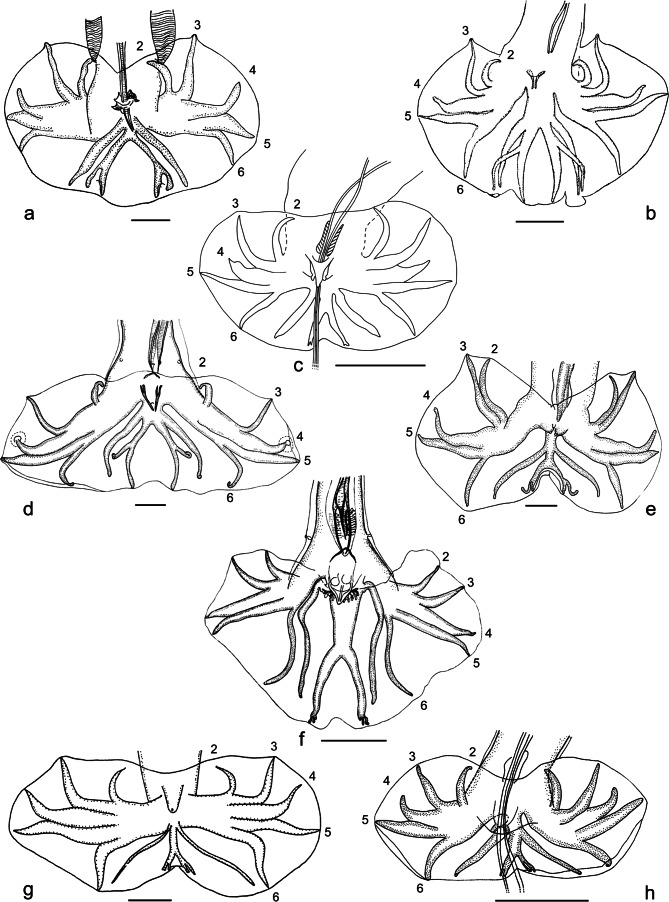
**Figs. 3a-d** Basic patterns. Scale-bars: 50 μm. a. Type 2-3. Rays 2 and 3 are grouped, arising first and together from the common trunk of rays 2 to 6; rays 4 to 6 have a common trunk and diverge at the same level. Ex: *Paraheligmonella interrogans* (Lent & Freitas, 1938). After Durette-Desset (1968b), modified. b. Type 2-2-1. Rays 2 and 3 are grouped, arising first and together from the common trunk of rays 2 to 6; ray 6 arises at the same level as ray 3; rays 4 and 5 are the last to diverge. Ex: *Heligmostrongylus crucifer* (Travassos, 1943). After Travassos (1943), modified. c. Type 1-3-1. Rays 2 and 6 arise first and at the same level from the common trunk of rays 2 to 6; rays 3 to 5 have a common trunk. Ex: *Hypocristata tercera* Durette-Desset & Guerrrero, 2006. In this case ray 3 diverges first from the common trunk of rays 3 to 5 in the right lobe and at the same level as ray 5 in the left lobe. After Durette-Desset & Guerrrero (2006), modified. d. Type 1-4. Ray 2 arises first from the common trunk of rays 2 to 6; rays 3 to 6 have a long common trunk. Ex: *Fuellebornema bocqueti* (Durette-Desset, 1970a). In this case ray 3 diverges at the same level as ray 6 on the common trunk of rays 3 to 6. After Durette-Desset (1970a), modified. **Figs. 3e-h** Intermediary patterns. Scale-bars: 50 μm. e. Type 2-3 t 2-2-1. Rays 2 and 3 are grouped, arising first and together from the common trunk of rays 2 to 6; rays 4 to 6 have a common trunk; ray 6 diverges close to the level of the divergence of ray 3 and proximally to that of rays 4 and 5. Ex: *Neoheligmonella acomysi* Durette-Desset & Gibson, 1990. In this case rays 2 and 3 are apposed for much of their length. After Durette-Desset & Gibson (1990), modified. f. Type 2-2-1 t 1-3-1. Rays 2 and 6 arise first and at the same level from the common trunk of rays 2 to 6; rays 3 arise just distally to the level of divergence of rays 2 and 6; rays 4 and 5 are the last to diverge. Ex: *Pudica pudica* (Travassos, 1921). After Cassone & Durette-Desset (1991), modified. g. Type 1-3-1 t 1-4. Rays 2 arise first from the common trunk of rays 2 to 6; rays 6 arise slightly distally to the level of divergence of rays 2 and proximally to that of rays 3; rays 3 to 6 have a short common trunk. Ex: *Spalacina yanchevi* Biserkov, Durette-Desset & Genov, 1995. After Biserkov *et al.* (1995), modified. h. Type 2-2-1 t 1-4. Rays 2 arise first from the common trunk of rays 2 to 6; rays 3-6 have a short common trunk; rays 6 arise at the same level as rays 3; rays 4 and 5 are the last to diverge. Ex: *Hypocristata anguillula* (Durette-Desset, 1971). Ray 3 is still grouped with ray 2 since its extremity supports the ventral lobe. After Durette-Desset (1971), modified.
- Intermediary patterns (or transitional types from a basic type to another): rays 2 to 6 show an intermediary arrangement with features of two basic types: type 2-3 tending to type 2-2-1 (2-3 t 2-2-1) (Fig. 3e); type 2-2-1 tending to type 1-3-1 (2-2-1 t 1-3-1) (Fig. 3f); type 1-3-1 tending to type 1-4 (1-3-1 t 1-4) (Fig. 3g); type 2-2-1 tending to type 1-4 (2-2-1 t 1-4) (Fig. 3h).

- Basic patterns: rays 2 to 6 show a characteristic arrangement of the following types: type 2-3 (Fig. 3a); type 2-2-1 (Fig. 3b); type 1-3-1 (Fig. 3c); type 1-4 (Fig. 3d).

### Literature data

The data were compiled from descriptions published in the literature. We took into account only the caudal bursae where both latero-ventral lobes, or at least one, was spread out. In the latter case, the lobe was treated separately and the pattern was not extrapolated to the entire bursa.

The systematic position of some species remains uncertain since, even if the caudal bursa is completely described, this is not the case with the synlophe. These species have therefore not been considered in this work. They are as follows: *Durettestrongylus travassosi* (Lent & Freitas, 1938), *Heligmonella vladimiri* Sadovskaja, 1952, *Heligmonina vogeli* Khalil, 1931, *Heligmonoides crassidorsualis* Franco, 1967, *Heligmonoides mirzai* Smales, 2008, *Heligmosomum delta* Travassos, 1921, *Longistriata degusi* Babero & Cattan, 1975, *Longistriata castrosilvai* Almeida, 1934, *Longistriata fortuita* Freitas, Lent & Almeida, 1937, *Longistriata perfida* Travassos, 1943, *Gobindonema boodugi* Sood & Parshad 1974, *Morganiella cricetuli* Yin & Zhang, 1981. They are here considered as Heligmonellidae *incertae sedis* since their generic allocation is not possible. In the particular case of *H. crassidorsualis* and *H. mirzai*, both were considered as Nippostrongylinae *incertae sedis* by Durette-Desset & Digiani (2010). Another species not treated is *Nesomystrongylus fissicauda* Durette-Desset, Lehtonen & Haukisalmi, 2002. This species shares more characters with the Heligmosomidae than with the Heligmonellidae, *i.e.* presence of caudal spine in the female tail, caudal bursa with rays 2 and 3 well developed, small dorsal lobe, and axis of orientation of synlophe subfrontal. It is likely that the monospecific genus *Nesomystrongylus* Durette-Desset, Lehtonen & Haukisalmi, 2002 should be transferred to the Heligmosomidae.

## Results

### Description of the patterns

#### • Basic and intermediary types

- Type 2-3 (Fig. 3a)
  - Rays 2 and 3 grouped, arising first and together from common trunk of rays 2 to 6.
  - Rays 2 and 3 grouped from base in V-shape.
  - Rays 4 to 6 having common trunk.
  - Divergence of rays 4, 5 and 6 at same level.
- Type 2-3 t 2-2-1 (*e.g.* type 2-3 tending to type 2-2-1, see Material & Methods) (Fig. 3e)
  - Rays 2 and 3 grouped, arising first and together from common trunk of rays 2 to 6.
  - Rays 4 to 6 having common trunk.
  - Divergence of ray 6 proximal to divergence of rays 4 and 5 and approximately at same level of divergence of ray 3.
- Type 2-2-1 (Fig. 3b)
  - Rays 2 and 3 grouped, arising first and together from common trunk of rays 2 to 6.
  - Ray 6 arising at the same level as ray 3.
  - Distal divergence of rays 4 and 5.
- Type 2-2-1 t 1-3-1 (Fig. 3f)
  - Rays 2 and 6 arising first and at same level from common trunk of rays 2 to 6.
  - Ray 3 arising just distally to level of divergence of rays 2 and 6.
  - Distal divergence of rays 4 and 5.
- Type 1-3-1 (Fig. 3c)
  - Rays 2 and 6 arising first and at same level from common trunk of rays 2 to 6.
  - Rays 3 to 5 having common trunk.
- Type 1-3-1 t 1-4 (Fig. 3g)
  - Ray 2 arising first from common trunk of rays 2 to 6.
  - Ray 6 arising slightly distally to level of divergence of ray 2 and proximally to level of divergence of ray 3.
  - Rays 3 to 6 having short common trunk.
- Type 1-4 (Fig. 3d)
  - Ray 2 arising first from common trunk of rays 2 to 6.
  - Rays 3 to 6 having long common trunk.
- Type 2-2-1 t 1-4 (Fig. 3h)
  - Ray 2 arising first from common trunk of rays 2 to 6.
  - Ray 6 arising at the same level as ray 3.
  - Rays 3 to 6 having short common trunk.
  - Distal divergence of rays 4 and 5.

##### Remarks

**Fig. 4:**
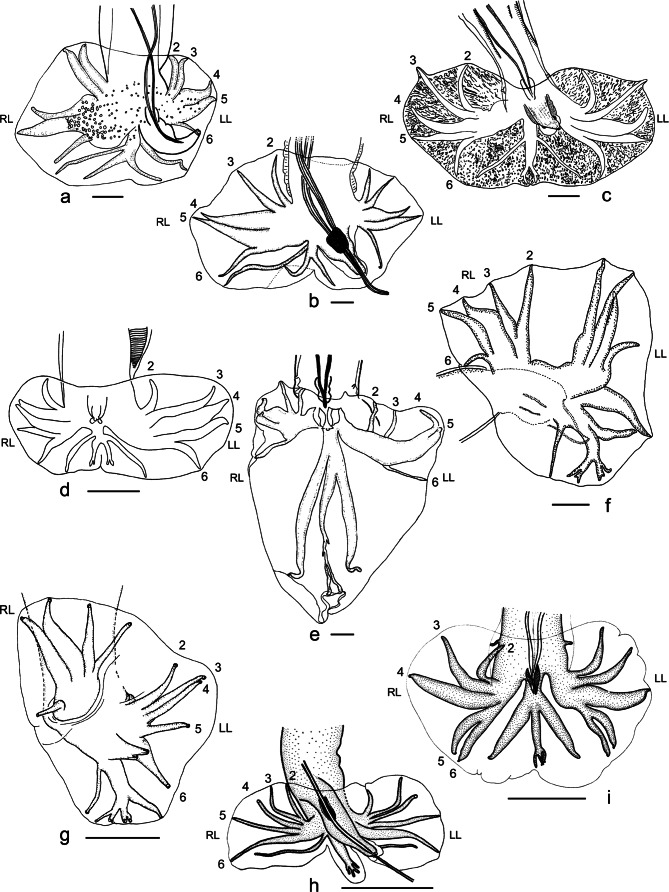
**Figs. 4a–d** Variations on types 2-2-1, 1-3-1 and 1-4. Scale-bars: 50 μm. a. Type 2-2-1 with rays 2 and 3 apposed for much of their length. Ex: *Stilestrongylus freitasi* Durette-Desset, 1968a. After Durette- Desset (1968a), modified. b. Type 2-2-1 with rays 2 and 3 grouped from the base in a narrow V-shape. Ex: right lobe of *Stilestrongylus andalgala* Digiani & Durette-Desset, 2007. After Digiani & Durette-Desset (2007), modified. c. Type 2-2-1 with rays 2 and 3 grouped from the base in a wide V-shape. Ex: *Carolinensis kinsellai* (Durette-Desset, 1969). After Durette-Desset (1969), modified. d. Type 1-3-1 with rays 3 diverging distally to rays 5 from the common trunk of rays 3 to 5. Ex: right lobe of *Heligmonina wakelini* Durette-Desset, Digiani, Mahlaba & Behnké, 2007. After Durette- Desset *et al.* (2007), modified. **Figs. 4e–i** Atypical patterns. Scale-bars: 50 μm. e. Type 1-1-2-1. Rays 2 arise first from the common trunk of rays 2 to 6; rays 3 are completely separated and parallel to rays 2 and arise at the same level as rays 6; rays 4 and 5 are the last to diverge. Ex: left lobe of *Cordicauda cordicauda* (Durette-Desset, 1966). After Durette-Desset (1966), modified. f. Type 1-3-1 t 4-1. Ray 6 arises first from the common trunk of rays 2 to 6; ray 2 arises just distally to the level of divergence of ray 6 and rays 2 to 5 have a short common trunk. Ex: right lobe of *Nippostrongylus magnus* (Mawson, 1961). Type 4-1. Ray 6 arises first from the common trunk of rays 2 to 6 and rays 2 to 5 have a long common trunk. Ex: left lobe of *N. magnus*. After Beveridge & Durette-Desset (1992), modified. g. Type 3-1-1. Rays 5 and 6 arise first but separately from the common trunk of rays 2 to 6 and rays 2 to 4 have a long common trunk. Ex: left lobe of *Nippostrongylus marhaeniae* Hasegawa & Syafruddin, 1995. The right lobe shows a pattern of type 1-3-1 t 4-1. After Hasegawa & Syafruddin (1995), modified. h. Type 1-2-1-1. Ray 2 arises first from the common trunk of rays 2 to 6; rays 3 and 4 are grouped and rays 5 and 6 arise separately and at the same level as the group formed by rays 3 and 4. Ex: left lobe of *Sciuricola moreli* (Gibbons, Durette-Desset & Daynes, 1977). The right lobe shows a pattern of type 2-2-1. After Durette- Desset (1974), modified. i. Type 1-2-2. Ray 2 arises first from the common trunk of rays 2 to 6; rays 3 and 4 are grouped; rays 5 and 6 have a short common trunk and arise at the same level as ray 2 and the group formed by rays 3 and 4. Ex: right lobe of *Trichoslinstowia maseri* Durette- Desset & Vaucher, 1974. After Durette-Desset & Vaucher (1974), modified. Type 2-1-2. Rays 2 and 3 are grouped and arise first from the common trunk of rays 2 to 6; ray 4 is isolated; rays 5 and 6 have a short common trunk and arise at the same level as ray 4 and the group formed by rays 2 and 3. Ex: left lobe of *T. maseri*. After Durette-Desset & Vaucher (1974), modified.

In the patterns where rays 2 and 3 are grouped, the grouping shows different types: rays 2 and 3 may be joined to a lesser (Fig. 3e) or greater extent (Fig. 4a) or having a V-shape with the branches separated to a lesser (Fig. 4b) or greater extent (Fig. 4c). In the latter case, rays 2 and 3 may be very distant from each other at their extremity, however both rays are all the same considered as grouped with rays 3 still supporting the ventral lobe. Such cases can be included in type 2-2-1 (Fig. 4c) or 2-2-1 t 1-4 (Fig. 3h), depending respectively on the presence or the absence of a short common trunk of rays 3 to 6.

In type 1-3-1, on the common trunk of rays 3 to 5, ray 3 can diverge proximally to (Fig. 3c, RL), at same level as (Fig. 3c, LL), or distally to (Fig. 4d, RL) ray 5.

In type 1-4, on the common trunk of rays 3 to 6, ray 3 can diverge proximally to (Fig. 3c, RL), at same level as (Fig. 3d, LL), or distally to (Fig. 4d, LL) ray 6.

#### • Atypical patterns

Seven atypical patterns were found among the Heligmonellidae:

- Type 1-1-2-1 (Fig. 4e, LL)
  - Ray 2 arising first from common trunk of rays 2 to 6.
  - Ray 3 completely separated and parallel to ray 2, arising at the same level as ray 6.
  - Distal divergence of rays 4 and 5.
- Type 1-3-1 t 4-1 (Fig. 4f, RL)
  - Ray 6 arising first from common trunk of rays 2 to 6.
  - Ray 2 arising just distally to level of divergence of ray 6.
  - Rays 2 to 5 having very short common trunk.
- Type 4-1 (Fig. 4f, LL)
  - Ray 6 arising first from common trunk of rays 2 to 6.
  - Rays 2 to 5 having long common trunk.
- Type 3-1-1 (Fig. 4g, LL)
  - Rays 5 and 6 arising first but separated from common trunk of rays 2 to 6.
  - Rays 2 to 4 having long common trunk.
- Type 1-2-1-1 (Fig. 4h)
  - Ray 2 arising first from common trunk of rays 2 to 6.
  - Rays 3 and 4 grouped.
  - Rays 5 and 6 arising separated and at same level as group formed by rays 3 and 4.
- Type 1-2-2 (Fig. 4i, RL)
  - Ray 2 arising first from common trunk of rays 2 to 6.
  - Rays 3 and 4 grouped.
  - Rays 5 and 6 having short common trunk, arising at the same level as ray 2 and group formed by rays 3 and 4.
- Type 2-1-2 (Fig. 4i, LL)
  - Rays 2 and 3 grouped and arising first from common trunk of rays 2 to 6.
  - Ray 4 isolated.
  - Rays 5 and 6 having short common trunk, arising at the same level as ray 4 and group formed by rays 2 and 3.

### Evolutionary interpretation of the patterns

**Fig. 5:**
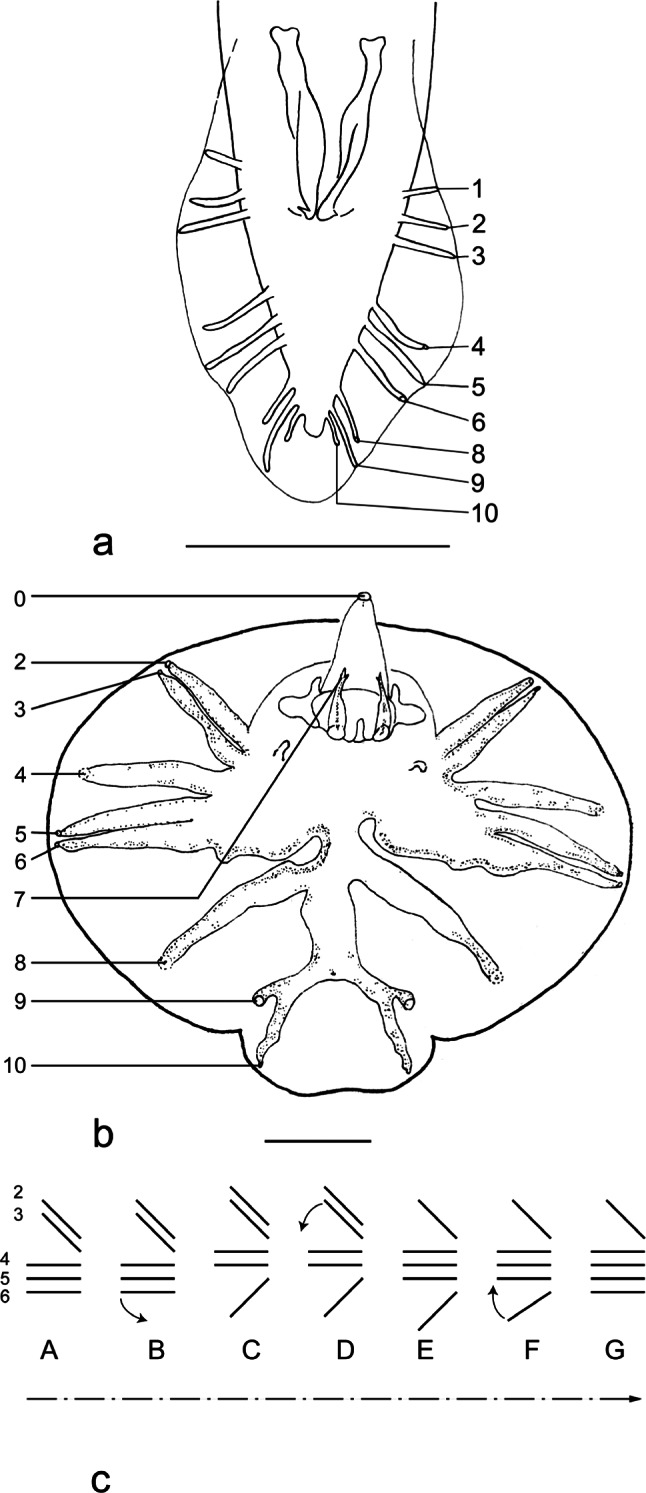
**Figs. 5a-b** Homology of the bursal rays of the Rhabditida and the Strongylida. Scale-bars: 50 μm. a. Bursal rays of the Rhabditida *sensu* Osche (1958). Papillae 7 are fused with papillae 8. After Osche (1958), modified. b. Bursal rays of the Strongylida *sensu* Chabaud *et al*. (1970). Papillae 7 are situated on the genital cone. After Chabaud *et al.* (1970), modified. **Fig. 5c** Schematic representation of the bursal patterns of rays 2 to 6 showing the hypothetical evolutionary steps. From the basal pattern of type 2-3, the evolutionary steps follow the direction: 2-3 tending to 2-2-1 to 1-3-1 to 1-4 with the intermediary patterns: 2-3 tending to 2-2-1, 2-2-1 tending to 1-3-1, 1-3-1 tending to 1-4. Some lobes have a pattern of type 2-2-1 t 1-4. A. Type 2-3. B. Type 2-3 t 2-2-1. C. Type 2-2-1. D. Type 2-2-1 t 1-3-1. E. Type 1-3-1. F. Type 1-3-1 t 1-4. G. Type 1-4. The small arrows indicate the transition from a type to another. The dotted arrow indicates the hypothetic evolutionary progress of the pattern of the caudal bursae.

Durette-Desset (1985) highlighted two evolutionary trends treating the Strongylida as a set: (1) reduction of the dorsal lobe (in the Rhabditida the tail of the male is vertically elongated) and (2) lengthening of rays 4, as papillae 4 of the Rhabditida are close to the anus (see Osche 1958; Chabaud *et al.*, 1970 for the homology between the papillae of the Rhabditida and the bursal rays of the Strongylida) (Figs. 5a, 5b). Therefore Durette-Desset (1985) suggested that a caudal bursa with a short dorsal lobe and long rays 4 should be considered as highly evolved. Though these trends occur in each type of bursa, in general, types 2-1-2 and 2-3 (among the five types of pattern recognized) were considered as basal types for two reasons: they are the closest to the pattern of the Rhabditida with a well developed dorsal lobe and short rays 4; and they are also present in all four suborders of the Strongylida. However, no further attempt was made to explain the other patterns from an evolutionary point of view. We propose the following interpretation (Fig. 5c).

In the Heligmonellidae the most basal pattern found is of type 2-3 (Fig. 3a). In this type, the dorsal lobe is relatively long as is ray 6, which diverges distally from the common trunk of rays 4 to 6.

The transition from type 2-3 to type 2-2-1 involves the migration of ray 6 towards the base of the trunk of rays 2 to 6, as seen in the intermediary type 2-3 t 2-2-1 (Fig. 3e). In type 2-2-1 (Fig. 3b), ray 6 has achieved this migration and diverges from the common trunk at same level as rays 3.

The transition from type 2-2-1 to type 1-3-1 involves a progressive migration of ray 3 towards ray 4 along with a progressive shortening of the dorsal lobe and ray 6, as observed in the intermediary type 2-2-1 t 1-3- 1 (Fig. 3f). In type 1-3-1 (Fig. 3c), the migration of ray 3 towards rays 4 is achieved, with the appearance of a common trunk of rays 3-5, and ray 6 diverges from this common trunk at same level as ray 2.

The transition from type 1-3-1 to type 1-4 involves a distal migration of ray 6, with the appearance of a common trunk of rays 3 to 6, and a progressive lengthening of this trunk, as observed in the intermediary type 1-3-1 t 1-4 (Fig. 3g). In type 1-4 (Fig. 3d), only ray 2 arises first from the common trunk. This type of caudal bursa widens laterally and its width is greater than its length.

The evolutionary steps then seem to follow the direction: 2-3 to 2-2-1 to 1-3-1 to 1-4, which is consistent with the early interpretation by Durette-Desset (1985) with respect to the basal patterns and the caudal bursae in which the width is greater than the length. Several descriptions of caudal bursae, especially in species of *Heligmonina* where the pattern was described as being of type 1-4 tending to type 1-3-1 (see for example Diouf *et al.*, 2005) are therefore inaccurate from an evolutionary point of view.

Some lobes may show a pattern of intermediary type 2-2-1 t 1-4 (Fig. 3h). This transition from type 2-2-1 involves the lengthening from the base of the set of rays 3-6 just after the divergence of ray 2, with the appearance of a short common trunk of rays 3 to 6 (intermediary type 2-2-1 t 1-4).

A particular 2-3 type was observed in some bursae or isolated lobes of the genera *Srivastavanema* (Singh, 1962) (Brevistriatinae) and *Malvinema* Digiani, Sutton & Durette-Desset, 2003 (Nippostrongylinae). In these cases one or both lateral lobes show an arrangement of rays 2 to 6 corresponding to a type 2-3, however the dorsal lobe is greatly reduced, the caudal bursa is laterally elongated and they are typically complementary with type 1-4. This means that in two species of *Malvinema*, *M. carolinae* Digiani, Sutton & Durette-Desset, 2003 and *M. victoriae* Digiani, Sutton & Durette-Desset, 2003, whereas one lobe is of type 2-3, the other lobe shows a pattern of type 1-4 (Figs. 2c, 2d); the remaining two species, *M. scapteromys* (Suriano & Navone, 1996) and *M. yagoi* Digiani & Durette-Desset, 2003, show the type 1-4; in two species of *Srivastavanema*, *S. cynocephali* Durette-Desset & Purwaningsih, 1999 and *S. yapi* Durette-Desset & Lim Boo Liat, 1975, both lobes are of type 2-3 whereas a third species *S. bhagwansinghi* Durette-Desset & Lim Boo Liat, 1975 shows a type 1-4 in both lobes. We thus consider this type 2-3 as different from that of the most basal species. It may be derived from the type 1-4 by the migration of ray 3 towards ray 2, whereas rays 4 to 6 still have a long common trunk.

#### • Atypical patterns

Type 1-1-2-1 (Fig. 4e) is present in the left lobe of *Cordicauda cordicauda* (Durette-Desset, 1966) (Brevistriatinae). This type seems to have been derived from type 2-2-1 by the isolation of ray 2 and short migration of ray 3 on the common trunk of rays 2 to 6.

Type 1-3-1 t 4-1 (Fig. 4f, RL) is present in the right lobes of *Nippostrongylus magnus* (Mawson, 1961) and *N. marhaeniae* Hasegawa & Syafruddin, 1995 (Nippostrongylinae). The transition from type 1-3-1 to type 4-1 involves a distal migration of ray 2 towards ray 3, forming a short common trunk of rays 2 to 5. Ray 6 is the most proximal ray to diverge from the common trunk of rays 2-6 and ray 2 arises just slightly distally to ray 6.

Type 4-1 (Fig. 4f, LL) is present in both lobes of *Nippostrongylus sembeli* Hasegawa & Tarore, 1995; and in the left lobes of *N. magnus*, *N. rauschi* Chabaud & Desset, 1966 and *N. typicus* (Mawson, 1961). This type seems directly derived from type 1-3-1, involving a marked distal migration of ray 2 towards ray 3. Ray 6 is the most proximal ray to diverge from the common trunk of rays 2-6, and rays 2 to 5 form a long common trunk on which ray 2 arises distinctly distally.

Type 3-1-1 (Fig. 4g, LL) is present in the left lobes of four species of *Nippostrongylus*: *N. djumachani* (Tenora, 1969), *N. marhaeniae*, *N. rysavyi* (Erhardova, 1959) and *N. witenbergi* Greenberg, 1972. It seems directly derived from type 4-1 by the migration of ray 5 from its base towards ray 6. Ray 5 diverges from the common trunk at the same level as ray 6 and there is persistence of the common trunk of rays 2 to 4.

Type 1-2-1-1 (Fig. 4h, LL) is present in the left lobes of *Sciuricola dremomys* (Yen, 1973) and *S. moreli* (Gibbons, Durette-Desset & Daynes, 1977) (Heligmonellinae). It seems directly derived from type 1-3-1 by the migration (but not apposition) of ray 5 towards ray 6, with persistence of the group formed by rays 3 and 4.

Type 1-2-2 (Fig. 4i, RL) is present in the right lobe of *Tricholinstowia maseri* Durette-Desset & Vaucher, 1974 (Heligmonellinae). It seems to be derived from type 1- 3-1 (present in the other species of *Tricholinstowia*) by the migration of ray 5 towards ray 6. Type 2-1-2 (Fig. 4i, LL) is present in the left lobe of the same species. It could be derived from type 1-2-2 by the migration of ray 3 towards ray 2, along with the absence of common trunk between rays 3 and 4.

A pattern of type 2-1-2 is characteristic of the Molineoidea. However, type 2-1-2 of the Molineoidea is interpreted as a basal pattern, also characterized by short rays 4, whereas the pattern found in *T. maseri*, with long rays 4, is considered as derived from a 1-3- 1 type. In this case, we consider the presence of this type in one lobe of Heligmonellidae as a convergence.

### Criteria utilized for the characterization of the caudal bursae of the heligmonellidae

#### • Type of symmetry (definition and examples above)

At the specific level, a caudal bursa may be subsymmetrical (CB SS); dissymmetrical with right lobe larger (CB DS RL+); dissymmetrical with left lobe larger (CB DS LL+); asymmetrical (CB AS); asymmetrical with right lobe larger (CB AS RL+); asymmetrical with left lobe larger (CB AS LL+). At the generic level, we consider that a given genus may be characterized by the type of symmetry most frequently found among the species belonging to this genus.

#### • Evolutionary comparison between lobes

As mentioned above, a species can have the same or a different pattern in both latero-ventral lobes. On the other hand, as seen above, the different patterns may be interpreted from an evolutionary point of view. This determines three types of “evolutionary ratio” between both lateral lobes (“right/left ratio”) in a caudal bursa: (1) the same pattern in both lobes; (2) the pattern of the right lobe is derived with respect to that of the left lobe (“RL derived”); or (3) the pattern of the left lobe is derived with respect to that of the right lobe (“LL derived”).

#### • Characteristic pattern

Within a given genus the bursal pattern usually varies among the species. Moreover, as seen immediately above, most species have a different pattern in each lobe. This means that in some genera we can find several different patterns. However it seems possible to choose one or two characteristic patterns for each genus. The characteristic pattern is determined by the type most frequently found, which is here interpreted as the most frequent basic type plus the contiguous intermediary types. For example, for the genus *Sciurodendrium* Durette-Desset, 1971 (Pudicinae, seven species) we propose that the characteristic pattern is of type 2-2-1 because amongst the 11 treatable lobes, four are of type 2-2-1, three of type 2-3 t 2-2-1, and two of type 2-2-1 t 1-3-1. Some genera may have two characteristic patterns. For example in *Heligmonina* (Nippostrongylinae, 27 species) two characteristic patterns are proposed: 1-3-1 and 1-4; type 1-3-1 being the most frequent in the right lobe and type 1-4 in the left lobe (21 and 19 lobes respectively).

### Characterization of the caudal bursae of the genera of the heligmonellidae

Heligmonellidae (Skrjabin & Schikhobalova, 1952, tribe) Durette-Desset, 1971 (four subfamilies, 56 genera, 329 species; for each genus, in parentheses, number of species examined / number of species described)
Heligmonellinae (Skrjabin & Schikhobalova, 1952, tribe) Durette-Desset & Chabaud, 1977 (six genera, 22 species)
  - *Heligmonella* Mönnig, 1927 (3/4)

CB SS (2 spp.) or CB DS RL+ (1 sp.). CB with the same pattern in both lobes (2 spp.) or with LL derived (1 sp.). Patterns observed: 2-3 t 2-2-1, 2-2-1, 1-4. Characteristic pattern: 2-2-1.

1. *Paraheligmonella* Durette-Desset, 1971 (4/5)

CB SS. CB with same pattern in both lobes (3 spp.) or with RL derived (1 sp.). Patterns observed: 2-3, 2-3 t 2-2-1, 2-2-1, atypical 2-2-1 t 4-1. Characteristic pattern: 2-2-1.

Remark: the species *Paraheligmonella cubaensis* (Pérez Vigueras, 1943), with the pattern 1-4 in both lobes was considered with reservations as belonging to *Paraheligmonella* (Digiani *et al.*, 2009).

1. *Sciuricola* Durette-Desset, 1983 (2/2)

CB SS. CB with different patterns in both lobes (1 sp.). Patterns observed: 2-3 t 2-2-1, atypical 1-2-1-1. Characteristic pattern: the data are insufficient to choose a characteristic pattern but two characters of the CB shared by both species are rays 4 and 5 separated from base and dorsal lobe distinct.

1. *Tricholinstowia* Travassos, 1937 (8/8)

CB SS (6 spp.) or CB DS LL+ (1 sp.), type of symmetry unknown in 1 sp. CB with the same pattern in both lobes (4 spp.) or with RL derived (1 sp.) or with LL derived (1 sp.), right/left ratio unknown in 2 spp. Patterns observed: 2-3 t 2-2-1, 2-2-1, 2-2-1 t 1-3-1, 1-3-1, atypical 1-2-2 and 2-1-2. Characteristic pattern: 1-3-1; rays 5 and 6 pincer-shaped.

Remark: only the right lobe is treatable in *T. mogera* (Sadovskaja, 1952) and *T. morenishi* (Cameron & Parnell, 1933).

1. *Trichotravassosia* Lent & Freitas, 1938 (2/2)

CB SS. CB with the same pattern in both lobes. Patterns observed: 2-2-1 t 1-4, atypical 2-2-1 t 4-1. Characteristic pattern: 2-2-1.

Remark: in *T. capromydis* Baruš & Rysavý, 1967 only the right lobe is treatable, showing an atypical pattern of type 2-2-1 tending to 4-1.

1. *Xericola* Durette-Desset, 1974 (1/1)

CB SS. CB with the same pattern in both lobes. Patterns observed: 2-2-1 t 1-4. Characteristic pattern: 2-2-1.

Brevistriatinae Durette-Desset, 1971 [12 genera (11 treated), 66 species]
  1. *Brevistriata* Travassos, 1937 (3/4)

CB SS. CB with the same pattern in both lobes. Patterns observed: 2-3 t 2-2-1, 2-2-1. Characteristic pattern: 2-2-1.

Remark: the caudal bursa of *B. fukiensis* Wang, Zao & Chen, 1978, is not included as the published illustration is not of a fully opened bursa.

1. *Calypsostrongylus* Schmidt, Myers & Kuntz, 1967 (7/7)

CB SS (5 spp.) or CB DS RL+ (2 spp.). CB with the same pattern in both lobes (5 spp.) or with LL derived (2 spp.). Patterns observed: 2-3 t 2-2-1, 2-2-1, 2-2-1 t 1-4. Characteristic pattern: 2-2-1.

1. *Cordicauda* Durette-Desset, 1971 (5/5)

CB DS LL+ (3 spp.) or CB SS (2 spp.). CB with the same pattern in both lobes (3 spp.) or LL derived (2 spp.). Patterns observed: 2-3 t 2-2-1, 2-2-1, 1-4. Characteristic pattern: 2-2-1.

1. *Fissicauda* Durette-Desset & Krishnansamy, 1976 (6/6)

CB SS (3 spp.) or CB DS LL+ (2 spp.) or CB DS RL+ (1 sp.). CB with the same pattern in both lobes (3 spp.) or with RL derived (3 spp.). Patterns observed: 2-3 t 2-2-1, 2-2-1, 2-2-1 t 1-3-1, 1-3-1. Characteristic pattern: 2-2-1.

1. *Kuala* Durette-Desset & Krishnansamy, 1976 (2/2)

CB SS. CB with the same pattern in both lobes. Pattern observed: atypical type 1-2-2.

1. *Lagostrongylus* Fukumoto, Kamiya & Ohbayashi, 1986 (3/3)

CB SS. CB with the same pattern in both lobes. Patterns observed: 2-3 t 2-2-1. Characteristic pattern: 2-3 t 2-2-1.

1. *Macrostrongylus* Ow-Yang, Durette-Desset & Ohbayashi, 1983 (2/2)

CB SS. CB with the same pattern in both lobes. Patterns observed: 2-3 t 2-2-1, 2-2-1. Characteristic pattern: 2-2-1.

1. *Metheligmonella* Durette-Desset, 1971 (2/2)

CB SS. CB with the same pattern in both lobes. Patterns observed: 2-3 t 2-2-1. Characteristic pattern: 2-3 t 2-2-1.

1. *Paraheligmonina* (Ortlepp, 1939) (27/28)

CB SS (12 spp.) or with a slight left (8 spp.) or right (7 spp.) dissymmetry. CB with the same pattern in both lobes (18 spp.) or with LL derived (7 spp.) or with RL derived (2 spp.). Patterns observed: 2-3 t 2-2-1, 2-2-1, 1-3-1, 1-4, atypical 2-2-1 t 4-1 and 4-1. Characteristic patterns: 2-2-1, 1-3-1.

Remark: the caudal bursa of *P. trifurcata* (Baylis, 1928) is not included as the published illustration is not of a fully opened bursa.

1. *Quentinstrongylus* Durette-Desset, 1969 (1/1)

CB SS. CB with the same pattern in both lobes. Patterns observed: 1-3-1. Characteristic pattern: 1-3-1.

1. *Srivastavanema* (Singh, 1962) (3/5)

CB SS. CB with the same pattern in both lobes. Patterns observed: secondary 2-3, and 1-4. Characteristic pattern: 1-4.

Pudicinae (Skrjabin & Schikhobalova, 1952, tribe) Durette-Desset, 1971 (nine genera, 44 species)
  1. *Acanthostrongylus* Travassos, 1937 (1/1)

CB SS. CB with the same pattern in both lobes. Pattern observed: 1-4. Characteristic pattern: 1-4.

1. *Durettestrongylus* Guerrero, 1982 (2/3)

CB SS. CB with the same pattern in both lobes. Patterns observed: 2-2-1. Characteristic pattern: 2-2-1.

1. *Freitastrongylus* Gonçalves, Pinto & Durette-Desset (1/1)

CB SS. CB with the same pattern in both lobes. Pattern observed: 1-4. Characteristic pattern: 1-4.

1. *Fuellebornema* Travassos & Darriba, 1929 (6/7)

CB SS. CB with the same pattern in both lobes. Pattern observed: 1-4. Characteristic pattern: 1-4.

Remark: the caudal bursa of *F. almeidai* Travassos, 1937 is not included as the published illustration is not of a fully opened bursa.

1. *Heligmostrongylus* Travassos, 1917 (9/9)

CB SS. CB with the same pattern in both lobes (8 spp.) or with LL derived (1 sp.). Patterns observed: 2-2-1, 2-2-1 t 1-3-1, 1-3-1. Characteristic pattern: 2-2-1.

1. *Justinema* R’Kha & Durette-Desset, 1991 (3/3)

CB SS. CB with RL derived (2 spp.) or with the same pattern in both lobes (1 sp.). Patterns observed: 2-2-1, 2-2-1 t 1-3-1. Characteristic pattern: 2-2-1.

1. *Pseudoheligmosomum* Travasssos, 1937 (1/1)

CB AS LL+. CB with the same pattern in both lobes. Pattern observed: 2-3. Characteristic pattern: 2-3.

1. *Pudica* Travassos & Darriba, 1929 (12/12)

CB SS. CB with the same pattern in both lobes (11 spp.) or with RL derived (1 sp.). Patterns observed: 2-3 t 2-2-1, 2-2-1, 2-2-1 t 1-3-1, 1-3-1, 1-4. Characteristic patterns: 2-2-1, 1-3-1.

1. *Sciurodendrium* Durette-Desset, 1971 (6/7)

CB SS. CB with LL derived (3 spp.) or with the same pattern in both lobes (2 spp.), right/left ratio unknown in 1 sp. Patterns observed: 2-3 t 2-2-1, 2-2-1, 2-2-1 t 1-3-1, 1-3-1. Characteristic pattern: 2-2-1.

Remarks: the caudal bursa of *S. aripense* (Baylis, 1947) is not included as the published illustration is not of a fully opened bursa. Only the right lobe is treatable in *S. bravohollisae* Falcón-Ordaz & Lamothe-Argumedo, 2006.

Nippostrongylinae Durette-Desset, 1971 (29 genera, 197 species)
  1. *Bunomystrongylus* Hasegawa & Mangali, 1996 (2/2)

CB DS RL+ (1 sp.) and CB DS LL+ (1 sp.). CB with RL derived. Patterns observed: 2-3 t 2-2-1, 2-2-1, 1-3-1, 1-4. No characteristic pattern.

1. *Carolinensis* (Travassos, 1937) (12/12)

CB SS (7 spp.) or CB DS LL+ (4 spp.) or CB DS LL+ (1 sp.). CB with the same pattern in both lobes (9 spp.) or with RL derived (1 sp.) or with LL derived (1 sp.), right/left ratio unknown in one species. Patterns observed: 2-3 t 2-2-1, 2-2-1, 2-2-1 t 1-3-1, 1-3-1, 1-3-1 t 1-4, 1-4. Characteristic pattern: 2-2-1.

Remark: only the right lobe is treatable in *C. eothenomysi* Asakawa, Kamiya & Ohbayashi, 1986 and *C. huehuetlana* Falcón-Ordaz & Sanabria Espinosa, 1996, the left lobe being not spread out.

1. *Euzetoda* Elias & Durette-Desset, 2003 (1/1)

CB SS. CB with the same pattern in both lobes. Pattern observed: 2-3 t 2-2-1. Characteristic pattern: 2-3 t 2-2-1.

1. *Guerrerostrongylus* Sutton & Durette-Desset, 1991 (2/2)

CB SS. CB with the same pattern in both lobes. Patterns observed: 1-3-1. Characteristic pattern: 1-3-1.

1. *Hasanuddinia* Hasegawa & Syafruddin, 1994 (1/1)

CB SS. CB with RL derived. Patterns observed: 2-2-1 t 1-3-1, 2-2-1 t 1-4. Characteristic pattern: 2-2-1.

1. *Hassalstrongylus* Durette-Desset, 1971 (13/14)

CB SS (6 spp.) or DS RL+ (6 spp.) or DS LL+ (1 sp.). CB with the same pattern in both lobes (9 spp.) or RL derived (2 spp.) or LL derived (2 spp.). Patterns observed: 2-3 t 2-2-1, 2-2-1, 2-2-1 t 1-3-1, 1-3-1, 1-4. Characteristic patterns: 1-4 (type species), 2-2-1, 1-3-1. Remark: the caudal bursa of *H. mazzai* Freitas, Lent & Almeida, 1937 is not included as the published illustration is not of a fully opened bursa.

1. *Heligmonina* Baylis, 1928 (24/26)

CB DS LL+. CB with LL derived (21 spp.) or with the same pattern in both lobes (3 spp.). Patterns observed: 1-3-1, 1-3-1 t 1-4, 1-4. Characteristic patterns: 1-3-1 on right lobe, 1-4 on left lobe.

Remarks: the caudal bursa of *H. oenomyos* Baylis, 1928 is not illustrated and therefore untreatable. The left lobe of *H. cricetomyos* Baylis, 1928 is typical of the genus *Heligmonina i.e.* of type 1-4, but the right lobe is atypical with a type 2-2-1 not retained in the patterns observed.

1. *Heligmonoides* Baylis, 1928 (12/12)

CB DS LL+. CB with the same pattern in both lobes (6 spp.) or with RL derived (3 spp.) or LL derived (3 spp.). Patterns observed: 2-3 t 2-2-1, 2-2-1, 2-2-1 t 1- 3-1, 1-3-1, 1-4. Characteristic pattern: 2-2-1.

1. *Hypocristata* Durette-Desset, 1971 (3/3)

CB SS (2 spp.) or DS RL+ (1 sp.). CB with the same pattern in both lobes (1 sp.) or with RL derived (1 sp.) or with LL derived (1 sp.). Patterns observed: 2-2-1, 2-2-1 t 1-4, 2-2-1 t 1-3-1, 1-3-1. Characteristic pattern: 1-3-1.

1. *Malaistrongylus* Ow-Yang, Durette-Desset & Ohbayashi, 1983 (1/1)

CB SS. CB with the same pattern in both lobes. Pattern observed: 2-2-1. Characteristic pattern: 2-2-1.

1. *Malvinema* Digiani, Sutton & Durette-Desset, 2003 (4/4)

CB DS RL+ (3 spp.) or CB AS RL+ (1 sp.). CB with the same pattern in both lobes (2 spp.) or with RL derived (1 sp.) or with LL derived (1 sp.). Patterns observed: 1-4, secondary 2-3. Characteristic pattern: 1-4.

1. *Mammanidula* Sadovskaja, 1952 (3/5)

CB AS RL+ (2 spp.) or CB SS (1 sp.). CB with LL derived (2 spp.) or RL derived (1 sp.). Patterns observed: 2-3, 2-3 t 2-2-1, 2-2-1. Characteristic pattern: 2-2-1.

Remark: the caudal bursae of *M. melomyos* (Mawson, 1961) and *M. siamensis* Ohbayashi & Vajrasthiva, 1983 are not included as the published illustrations are not of fully opened bursae.

1. *Mawsonema* Smales & Heinrich, 2010 (1/1)

CB SS. CB with the same pattern in both lobes. Pattern observed: 2-3. Characteristic pattern: 2-3.

1. *Maxomystrongylus* Hasegawa & Syafruddin, 1997 (2/2)

CB DS LL+. CB with the same pattern in both lobes (1 sp.) or with RL derived (1 sp.). Patterns observed: 2-3, 2-3 t 2-2-1, 2-2-1. Characteristic pattern: 2-2-1.

1. *Melomystrongylus* Smales, 2009 (2/2)

CB DS LL+ or CB DS RL+. CB with RL derived (1 sp.), right/left ratio unknown in one species. Patterns observed: 2-3, 1-3-1. Characteristic pattern: 2-3.

Remark: only the left lobe of *M. sepikensis* Smales, 2009 is illustrated and treatable.

1. *Montistrongylus* Smales & Heinrich, 2010 (1/1)

CB DS RL+. CB with RL derived. Patterns observed: 2-3 t 2-2-1, 2-2-1. Characteristic pattern: 2-2-1.

1. *Neoheligmonella* Durette-Desset, 1971 (21/23)

CB SS. CB with the same pattern in both lobes (for at least 12 spp.) or with RL derived (for at least 7 spp.), right/left ratio unknown in 2 spp. Patterns observed: 2-3, 2-3 t 2-2-1, 2-2-1. Characteristic pattern: 2-3 t 2-2-1. Remarks: the caudal bursa of *N. affinis* (Baylis, 1928) is not treatable since it is not illustrated in the original description. Only the right lobe is treatable in *N. impudica* (Baylis, 1928) and *N. moennigi* (Baylis, 1928). *N. lemniscomysi* (Durette-Desset, 1970) is the only species having a caudal bursa with a right lobe larger and with the same pattern (1-3-1) in both lobes. On the other hand, its synlophe is also very different from that of the remaining species in the genus. It is likely that this species belongs to a different genus and it is not treated herein.

1. *Nippostrongylus* Lane, 1923 (9/9)

CB AS RL+ (6 spp.) or CB DS RL+ (3 spp.). CB with LL derived. Patterns observed: 1-3-1, 1-3-1 t 1-4, 1-3- 1 t 4-1, 4-1, 3-1-1. Characteristic patterns: 1-3-1 and derived atypical 4-1 on right lobe; atypical 4-1 and derived atypical 3-1-1 on left lobe.

1. *Odilia* Durette-Desset, 1973 (18/19)

CB SS (8 spp.) or DS RL+ (3 spp.) or DS LL+ (2 spp.), symmetry unknown in 5 spp. CB with same pattern in both lobes (at least 8 spp.) or with RL derived (at least 3 spp.), right/left ratio unknown in 5 spp. Patterns observed: 2-3, 2-3 t 2-2-1, 2-2-1, 2-2-1 t 1-3-1, 2-2-1 t 1-4, 1-3-1, 1-3-1 t 1-4, 1-4. Characteristic patterns: 2-2-1, 1-4.

Remarks: the caudal bursa of *O. polyrhabdote* (Mawson, 1961) is not included as the published illustration is not of a fully opened bursa. Only one lobe is treatable in *O. brachybursa* (Mawson, 1961), *O. implexa* Smales, 2008, *O. moatensis* (Hasegawa, Miyata & Syafruddin, 1999), *O. similis* Smales, 2009 and *O. uromyos* (Mawson, 1961).

1. *Orientostrongylus* Durette-Desset, 1970 (7/8)

CB SS. CB with the same pattern in both lobes (6 spp.) or with LL derived (1 sp.). Patterns observed: 2-3, 2-3 t 2-2-1, 2-2-1. Characteristic pattern: 2-2-1.

Remark: the caudal bursa of *O. siamensis* Ohbayashi & Kamiya, 1980 is not included as the published illustration is not of a fully opened bursa.

1. *Paraheligmonelloides* Fukumoto, Kamiya & Suzuki, 1980 (9/9)

CB DS LL+ (4 spp.) or CB DS RL+ (3 spp) or CB SS (2 spp.). CB with the same pattern in both lobes. Patterns observed: 2-3, 2-3 t 2-2-1, 2-2-1, 1-4. Characteristic patterns: 2-2-1, 1-4.

Remark: the caudal bursa of *P. singauwaensis* Smales, 2009 is treated from its redescription (Smales & Heinrich, 2010).

1. *Parasabanema* Smales & Heinrich, 2010 (1/1)

CB SS. CB with the same pattern in both lobes. Pattern observed: 2-2-1. Characteristic pattern: 2-2-1.

1. *Rattustr ongylus* Ow-Yang, Durette-Desset & Ohbayashi, 1983 (2/2)

CB SS (1 sp.) or CB DS LL+ (1 sp.). CB with the same pattern in both lobes. Patterns observed: 2-2-1, 2-2-1 t 1-3-1. Characteristic pattern: 2-2-1.

1. *Sabanema* Ow-Yang, Durette-Desset & Ohbayashi, 1983 (4/5)

CB DS RL+ (3 spp.) or CB SS (1 sp.). CB with the same pattern in both lobes (2 spp.) or with RL derived (1 sp.) or with LL derived (1 sp.). Patterns observed: 2-3 t 2-2-1, 2-2-1. Characteristic pattern: 2-2-1.

Remark: the caudal bursa of *S. kepongi* Ow Yang, Durette-Desset & Ohbayashi, 1983 is not included as the published illustration of the bursa is not of a fully opened bursa.

1. *Spalacina* Biserkov, Durette-Desset & Genov, 1995 (3/3)

CB SS (2 spp.) or DS LL+ (1 sp.). CB with the same pattern in both lobes (at least 2 spp.), right/left ratio unknown in one species. Patterns observed: 1-3-1 t 1-4, 1-4. Characteristic pattern: 1-4.

Remark: only the right lobe is treatable in *S. spalacis* (Sharpilo, 1973) the left lobe being not spread out.

1. *Stilestrongylus* Freitas, Lent & Almeida, 1937 (24/25)

CB DS RL+ (21 spp.) or CB SS (3 spp.). CB with RL derived (11 spp.) or with the same pattern in both lobes (10 spp.) or with LL derived (3 spp.). Patterns observed: 2-3 t 2-2-1, 2-2-1, 1-3-1, 1-4. Characteristic patterns: 2-2-1, 1-4.

Remark: the caudal bursa of *S. peromysci* Falcón-Ordaz & Sanabria-Espinoza, 1999 is not included as the published illustration is not of a fully opened bursa.

1. *Suttonema* Digiani & Durette-Desset, 2003 (1/1)

CB DS RL+. CB with the same pattern in both lobes. Pattern observed: 1-4. Characteristic pattern: 1-4.

1. *Trichofreitasia* Sutton & Durette-Desset, 1991 (1/1)

CB SS. CB with the same pattern in both lobes. Pattern observed: 2-2-1. Characteristic pattern: 2-2-1.

1. *Yatinema* Asakawa & Ohbayashi, 1985 (2/2)

CB SS (1 sp.) or CB DS RL+ (1 sp.). CB with RL derived (1 sp.), right/left ratio unknown in one species. Patterns observed: 2-2-1 t 1-4, 1-3-1. Characteristic pattern: 2-2-1.

Remark: only the right lobe of *Y. siamensis* Asakawa, Kamiya & Ohbayashi, 1986 is treatable, the left lobe being not spread out.

Based on the elements considered above, the four subfamilies may be synthetically characterized as follows: the Heligmonellinae by a subsymmetrical caudal bursa, with the same pattern in both lobes, and a characteristic pattern of type 2-2-1 (Table I); the Brevistriatinae by a predominantly subsymmetrical caudal bursa, a right/left ratio which is variable but with predominantly the same pattern in both lobes, and several types of pattern with a predominance of the characteristic type 2-2-1 (Table II); the Pudicinae by a subsymmetrical caudal bursa, a right/left ratio which is variable but with predominantly the same pattern in both lobes, and several types of patterns with a predominance of characteristic types 2-2-1 and 1-4 (Table III); the Nippostrongylinae by a caudal bursa of variable symmetry, a right/left ratio which is also variable and several types of pattern with a predominance of characteristic types 2-2-1 and 1-4 (Table IV).

**Table I:** Synopsis of the caudal bursa in the Heligmonellinae.

Genus	Characteristic symmetry	Right/left ratio	Characteristic pattern
*Heligmonella*	CB SS	Same pattern	2-2-1
*Paraheligmonella*	CB SS	Same pattern	2-2-1
*Sciuricola*	CB SS	LL atypical	-
*Tricholinstowia*	CB SS	Variable	1-3-1
*Trichotravassosia*	CB SS	Same pattern	2-2-1
*Xericola*	CB SS	Same pattern	2-2-1

CB SS: caudal bursa subsymmetrical. LL: left lobe of caudal bursa.

**Table II:** Synopsis of the caudal bursa in the Brevistriatinae.

Genus	Characteristic symmetry	Right/left ratio	Characteristic pattern
*Brevistriata*	CB SS	Same pattern	2-2-1
*Calypsostrongylus*	CB SS	Same pattern	2-2-1
*Cordicauda*	Variable	Variable	2-2-1
*Fissicauda*	Variable	Variable	2-2-1
*Kuala*	CB SS	Same pattern	1-2-2
*Lagostrongylus*	CB SS	Same pattern	2-3 t 2-2-1
*Macrostrongylus*	CB SS	Same pattern	2-2-1
*Metheligmonella*	CB SS	Same pattern	2-3 t 2-2-1
*Paraheligmonina*	Variable	Variable	2-2-1, 1-3-1
*Quentinstrongylus*	CB SS	Same pattern	1-3-1
*Srivastavanema*	CB SS	Same pattern	1-4

CB SS: caudal bursa subsymmetrical.

**Table III:** Synopsis of the caudal bursa in the Pudicinae.

Genus	Characteristic symmetry	Right/left ratio	Characteristic pattern
*Acanthostrongylus*	CB SS	Same pattern	1-4
*Durettestrongylus*	CB SS	Same pattern	2-2-1
*Freitastrongylus*	CB SS	Same pattern	1-4
*Fuellebornema*	CB SS	Same pattern	1-4
*Heligmostrongylus*	CB SS	Same pattern	2-2-1
*Justinema*	CB SS	Variable	2-2-1
*Pseudoheligmosomum*	CB DS LL+	Same pattern	2-3
*Pudica*	CB SS	Same pattern	2-2-1, 1-3-1
*Sciurodendrium*	CB SS	Variable	2-2-1

CB SS: caudal bursa subsymmetrical. CB DS LL+: caudal bursa dissymmetrical with right left lobe larger.

**Table IV:** Synopsis of the caudal bursa in the Nippostrongylinae.

Genus	Characteristic symmetry	Right/left ratio	Characteristic pattern
*Bunomystrongylus*	Variable	RL derived	-
*Carolinensis*	Variable	Same pattern	2-2-1
*Euzetoda*	CB SS	Same pattern	2-3 t 2-2-1
*Guerrerostrongylus*	CB SS	Same pattern	1-3-1
*Hasanuddinia*	CB SS	RL derived	2-2-1
*Hassalstrongylus*	Variable	Same pattern	1-4, 2-2-1, 1-3-1
*Heligmonina*	CB DS LL+	LL derived	1-3-1 RL, 1-4 LL
*Heligmonoides*	CB DS LL+	Variable	2-2-1
*Hypocristata*	CB SS	Variable	1-3-1
*Malaistrongylus*	CB SS	Same pattern	2-2-1
*Malvinema*	CB DS RL+	Variable	1-4
*Mammanidula*	CB AS RL+	Variable	2-2-1
*Mawsonema*	CB SS	Same pattern	2-3
*Maxomystrongylus*	BC DS LL+	Variable	2-2-1
*Melomystrongylus*	variable	Unknown	2-3
*Montistrongylus*	CB DS RL+	RL derived	2-2-1
*Neoheligmonella*	CB SS	Variable	2-3 t 2-2-1
*Nippostrongylus*	CB AS RL+	LL derived	1-3-1, 4-1 RL
	CB DS RL+		4-1, 3-1-1 LL
*Odilia*	Variable	Variable	2-2-1, 1-4
*Orientostrongylus*	CB SS	Same pattern	2-2-1
*Paraheligmonelloides*	Variable	Same pattern	2-2-1, 1-4
*Parasabanema*	CB SS	Same pattern	2-2-1
*Rattustrongylus*	Variable	Same pattern	2-2-1
*Sabanema*	CB DS RL+	Variable	2-2-1
*Spalacina*	CB SS	Same pattern	1-4
*Stilestrongylus*	CB DS RL+	Variable	2-2-1, 1-4
*Suttonema*	CB DS RL+	Same pattern	1-4
*Trichofreitasia*	CB SS	Same pattern	2-2-1
*Yatinema*	Variable	RL derived	2-2-1

CB SS: caudal bursa subsymmetrical. LL: left lobe of caudal bursa. RL: right lobe of caudal bursa. CB DS LL+: caudal bursa dissymmetrical with left lobe larger. CB DS RL+: caudal bursa dissymmetrical with right lobe larger. LL derived: left lobe of the caudal bursa having a pattern derived with respect to that of the right lobe. RL derived: right lobe of the caudal bursa having a pattern derived with respect to that of the left lobe.

## Discussion

Of the five main patterns recognized among the Trichostrongylina treated by Durette-Desset (1985), only three were observed in the Heligmonellidae: 2-3, 2-2-1 and 1-3-1. In this article, the main feature considered was the grouping of the rays and, in the interpretation of its evolution, only two main tendencies were considered: the shortening of the dorsal ray and the lengthening of rays 4.

In the present article, we provide an additional feature at the descriptive level, which is the sequence of the origin of the rays from the common trunk. Consequently, the presence of a new basic pattern (type 1-4) is highlighted, plus the presence of intermediary types: type 2-3 tending to 2-2-1, type 2-2-1 tending to 1-3-1, type 1-3-1 tending to 1-4 and type 2-2-1 tending to 1- 4, which have enabled us to attempt an evolutionary interpretation of the patterns.

Type 2-3, the basal type, is somewhat infrequent, but not the following intermediary type, 2-3 t 2-2-1, which is common and present in all four subfamilies. The basic type, 2-2-1, seems to be the most consistent pattern in the family: it is the most frequent characteristic pattern, is present in all four subfamilies, and there are relatively few intermediary types from 2-2-1 to 1-3-1, to 1-4 or to atypical patterns. Types 1-3-1 and 1-4 are less frequent than type 2-2-1 but are characteristic of several genera; type 1-3-1 is observed in all four subfamilies, and is the origin of several atypical types (4-1, 3-1-1, 1-2-2 and 1-2-1-1); type 1-4, characteristic of laterally elongated lobes, is absent from the Heligmonellinae. Finally, the family Heligmonellidae is characterized by the fact that, regardless of the pattern, with very few exceptions, rays 4 and 5 are always the last to diverge from the common trunk of rays 2 to 6.

It is clear that a dissymmetrical or asymmetrical caudal bursa should be regarded as derived with respect to a subsymmetrical one. However, the type of symmetry has been actually considered a character of little value above the species level, since the asymmetrical bursae and mostly the dissymmetrical bursae have arisen several times during the course of evolution. The dissymmetry usually involves the transverse (lateral) lengthening of one of the lobes. This dissymmetry probably plays a role in holding the female during copulation (Durette-Desset, 1985). The most conspicuous asymmetries and dissymmetries are found among the Nippostrongylinae. In species with a slight dissymmetry the lengthening may or may not modify the bursal pattern, which usually remains the same in both lobes. In species with strong dissymmetry, species, which are usually tightly coiled spirally, the pattern is usually different for each lobe.

Independent of the type of symmetry, both lobes of the caudal bursa may have the same or different patterns. In the latter case the most frequent situation from an evolutionary point of view is one lobe having one pattern and the other lobe showing the “next” intermediary or basic type (*e.g. Neoheligmonella* 2-3 t 2-2-1 and 2-2-1; *Heligmonina* 1-3-1 and 1-4); there are very few cases in which both lobes have a totally different pattern (*e.g. Heligmonella asymmetrica*, some species of *Stilestrongylus*).

In some genera the same “right/left ratio” is observed in all or most species in the genus: either both lobes have the same pattern (*Heligmostrongylus*, *Pudica*), or it is always the same lobe, which is derived with respect to the other (*Heligmonina*). Other genera are less homogeneous and the right/left ratio varies among the species of the genus (*Neoheligmonella*, *Paraheligmonina*

The combination of the right/left ratio and type of symmetry gives disparate results: in genera with marked dissymmetry or asymmetry, the hypertrophied lobe is usually the same but it may show either the derived pattern (*Heligmonina*, LL) or the basal pattern (*Nippostrongylus*, RL). In genera with subsymmetrical or slightly dissymmetrical caudal bursae, not only may the right/left ratio vary but also the type of symmetry within each genus (*Odilia*, *Paraheligmonina*).

It is interesting to note that the Heligmonellinae, the Brevistriatinae and the Pudicinae, in which most of the caudal bursae are subsymmetrical with the same pattern in both lobes, are parasitic in hosts, which are relatively ancient (mainly sciuromorph and caviomorph rodents, a few in insectivores and lagomorphs). The Nippostrongylinae, in which the symmetry and the pattern in both lobes are more variable, are parasitic in a group, which appeared more recently, the muroid rodents (mainly cricetids and murids).
